# A computational signature of self-other mergence in Borderline Personality Disorder

**DOI:** 10.1038/s41398-024-03170-w

**Published:** 2024-11-19

**Authors:** Giles W. Story, Sam Ereira, Stephanie Valle, Samuel R. Chamberlain, Jon E. Grant, Raymond J. Dolan

**Affiliations:** 1 Division of Psychiatry, University College London, London, UK; 2 Max Planck-UCL Centre for Computational Psychiatry and Ageing Research, University College London, London, UK; 3 Preventative Neurology Unit, Queen Mary University, London, UK; 4 Department of Psychiatry and Behavioral Neuroscience, University of Chicago, Chicago, IL USA; 5 Department of Psychiatry, Faculty of Medicine, University of Southampton, Southampton, UK; 6 Southern Health NHS Foundation Trust, Southampton, UK

**Keywords:** Human behaviour, Psychiatric disorders, Learning and memory, Diagnostic markers, Pathogenesis

## Abstract

A tendency to merge mental representations of self and other is thought to underpin the intense and unstable relationships that feature in Borderline Personality Disorder (BPD). However, clinical theories of BPD do not specify, in computational terms, how the perspectives of self and other might become confused. To address this question, we used a probabilistic false belief task (p-FBT) to examine how individuals with BPD (*N* = 38) and matched controls from the general population (*N* = 74) selectively assigned beliefs to self or other. The p-FBT requires participants to track a gradually changing quantity, whilst also predicting another person’s belief about that quantity. We found that BPD participants showed less selectivity in belief assignment compared with controls (Cohen’s *d* = 0.64). Behaviourally, participants with BPD tended to predict that others’ beliefs resembled their own. Modelling analysis revealed that BPD participants were prone to generalise their own learning signals to others. Furthermore, this generalising tendency correlated with BPD symptomatology across participants, even when controlling for demographic factors and affective psychopathology. Our results support a computational account of self-other mergence, based on a generalisation of learning across agents. Self-other generalisation in learning purports to explain key clinical features of BPD, and suggests a potential transdiagnostic marker of mentalising capability.

## Introduction

In Shakespeare’s play, ‘Romeo and Juliet’, Romeo takes his own life, believing Juliet to be dead. The audience, on the other hand, aware that Juliet has feigned her death, knows that Romeo is tragically mistaken. The dramatic effect rests on the audience’s ability to represent Romeo’s false belief, distinct from their own knowledge of the true state of affairs (see [1]). More broadly, this capacity to separately represent the mental states of self and other, termed ‘mentalising’, is fundamental to the effectiveness of our social interactions [2–9].

Limitations in mentalising are thought to underpin a syndrome of unstable self-identity and intense, brittle relationships, known as ‘Borderline Personality Disorder’ (BPD) [10–23]. This idea gains support in findings that people diagnosed with BPD exhibit reduced performance on false belief tasks [24–26], wherein subjects need to distinguish their own knowledge of a situation from that of a fictive observer, who has only partial information [27–29]. BPD participants also tend to perform less well than healthy controls in describing emotions from another person’s perspective [30–32], and in understanding when another person has made a *faux pas* (for instance, when a person reveals knowledge that ought to be hidden) [33–36]. These findings suggest that people with BPD have difficulty representing others’ mental states and distinguishing these from their own.

In clinical terms, a difficulty in subjectively separating mental states belonging to self and other putatively leads to a heightened vulnerability to others’ responses [14, 21–23]. For example, mentalising-based theories of BPD invoke a mode of ‘psychic equivalence’, wherein a person equates their own mental processes with external reality [2, 17–19]. Mental states then carry the force of objective facts, such that others are expected to know and share one’s thoughts and feelings. In such merged states, disagreement may be experienced, not merely as a difference in opinion or perspective, but as a confusing denial of what was assumed to be a shared reality [12, 20]. Theories across clinical traditions converge on a notion that such disturbance in the capacity to distinguish self from other predisposes to relationships that oscillate between intense closeness and precipitous rupture [12–16].

**Fig. 1:**
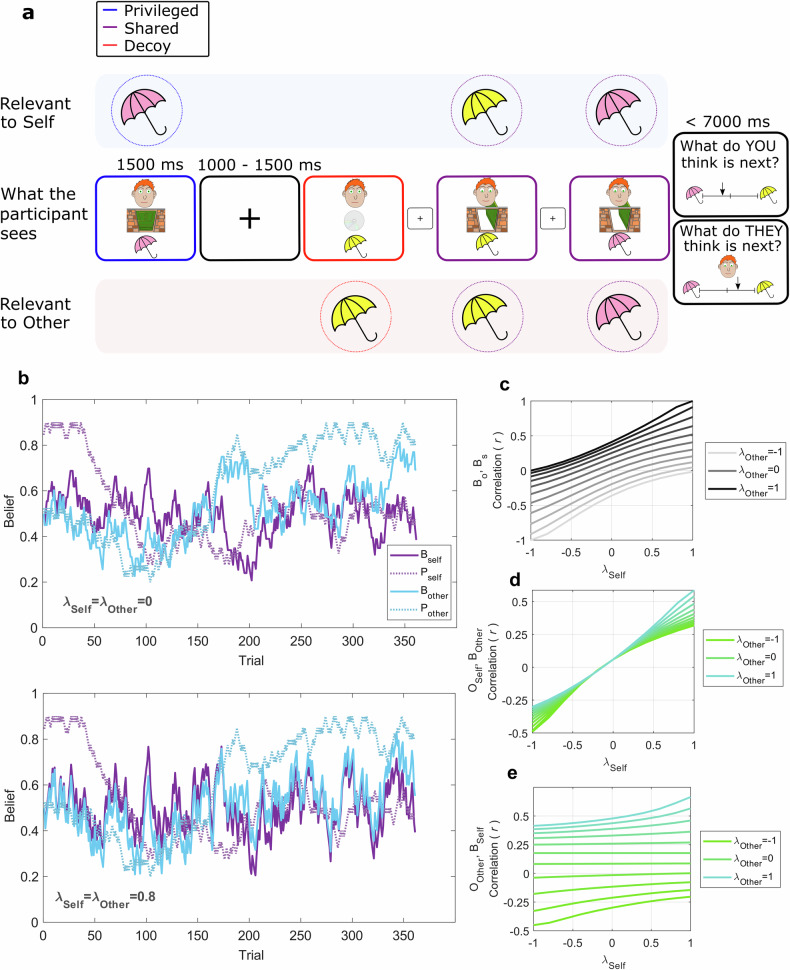
Probabilistic false-belief task (p-FBT). **a** Adapted from Ereira et al. (2020) [49]. Participants observed samples (purchases of pink umbrellas or yellow sunshades at a store) from a Bernoulli distribution. An hypothetical other person (the store manager), received partial or misleading information. On ‘Privileged’ trials, samples were hidden from the other person (shown as the store manager’s face behind a closed door). On ‘Shared’ trials the other person saw the same sample as the participant (shown as the manager’s face looking through an open door). On ‘Decoy’ trials the other person received a false sample (described as the manager’s watching out of date security camera footage, shown as the manager’s face above an image of a computer disc). Participants were intermittently asked to predict either the next sample, or the other person’s belief about the next sample, by placing a cursor on a continuous probability scale. **b** The underlying probability, *P*(umbrella), varied across time, with differing trajectories for the participant $({P}_{{{\rm {Self}}}})$ and other person $({P}_{{{\rm {Other}}}})$. Simulated belief trajectories are depicted for either fully agent-specific updating (${\lambda }_{{{\rm {Self}}}}={\lambda }_{{{\rm {Other}}}}=0$, $\alpha =0.1$, $\tau =0.04$ and $\delta =0$), or leakage in updating (${\lambda }_{{{\rm {Self}}}}={\lambda }_{{{\rm {Other}}}}=0$.8), showing that leakage induces a correlation in beliefs across agents. **c** Pearson correlation in beliefs for Self $({B}^{{{\rm {Self}}}})\,$ and Other (${B}^{{{\rm {Other}}}}$) derived from model simulation on the task with varying settings of ${\lambda }_{{{\rm {Self}}}}$ (horizontal axis) and ${\lambda }_{{{\rm {Other}}}}$ (solid lines) ($\alpha =0.1$, $\tau =0.04$ and $\delta =0$). **d** ${\lambda }_{{{\rm {Self}}}}\,$ induces a correlation between a recency-weighted average of outcomes pertinent to Other and ${B}^{{{\rm {Self}}}}$. **e** ${\lambda }_{{{\rm {Other}}}}$ induces correlation between outcomes pertinent to Self and ${B}^{{{\rm {Other}}}}$.

However, a shortcoming of existing theories is that they do not specify, in computational terms, how people selectively ascribe mental states to self or other. Nor do clinical theories explain how this selectivity might go awry in BPD. We address this by measuring belief updating using a novel probabilistic false belief task (p-FBT) (Fig. 1) [37, 38]. Previous false belief tasks classically use one-shot verbal or pictorial narratives wherein the beliefs of self and other are constrained to be either identical or opposite [24–26]. By contrast, the p-FBT requires participants to track a gradually changing quantity, whilst also predicting another person’s belief about that quantity. The p-FBT thereby allows beliefs attributed to self and other to develop along trajectories with varying degrees of correlation (Fig. 1b, c), enabling us to apply a model to interrogate the extent to which learning signals are selectively attributed to Self or Other. We administered the p-FBT in participants with diagnoses of BPD (*N* = 38) and in control participants recruited from the general population (*N* = 74), matched to the former on age (±4 years), gender and education.

Using computational modelling, we formalise a continuum between *agent-specific* updating, wherein beliefs of Self and Other are maintained separately, and *agent-invariant* updating, wherein Self and Other collapse to a single belief representation. We consider two directions of agent-invariant updating. Firstly, updating can be ‘idiocentric’, denoting the fact that one’s own learning signals generalise to others. Thus, events hidden from another person still influence one’s estimate of the other’s belief, akin to the mentalising errors seen in conventional false belief tasks [27–29]. On the p-FBT, this induces a correlation between estimates of the Other’s belief and outcomes observed by Self (Fig. 1d).

Secondly, updating can be ‘allocentric’, denoting generalisation of learning signals from Other to Self. Under allocentric updating, seeing another person observe false information nevertheless influences what the subject comes to believe about the world. Within the p-FBT, the subject sometimes sees the other person observe corrupted information. Allocentric updating induces a correlation between the subject’s belief and outcomes observed by the Other, even though these may be misleading (Fig. 1e).

Our central prediction was that participants with BPD would exhibit greater agent-invariant updating compared with participants sampled from the general population and that agent-invariant updating should relate to BPD symptom expression, as measured by the Borderline Symptom List [39]. We did not have strong prior hypotheses regarding whether BPD participants would tend to exhibit either idiocentric or allocentric updating.

## Materials and methods

### BPD participants

BPD participants were recruited to a randomised placebo-controlled trial situated at the University of Chicago, designed to assess the effect of an antipsychotic medication, brexpiprazole, in the treatment of BPD [40]. Recruitment took place between June 2018 and December 2020. Procedures were approved by the University of Chicago Institutional Review Board. The trial was registered with Clinicaltrials.gov (study identifier NCT03418675). Informed consent was obtained from all participants. Participants were compensated $200 for their participation, which entailed ten study visits.

A total of 80 BPD participants were recruited to the clinical trial, of which 38 completed the p-FBT. Data collection of the p-FBT was interrupted at the onset of the Covid-19 pandemic, when clinical trial follow-up moved online, at which point 29 participants had completed the task. The majority of BPD participants recruited to the trial after this point did not complete the p-FBT; nine BPD participants completed the task online, in a supervised online assessment. The experiment and instructions were identical for all participants regardless of whether performed online or in-person.

To be included in the trial, participants needed to be aged 18–65 years, have a primary diagnosis of BPD, and score at least nine points on the Zanarini rating scale for Borderline Personality Disorder (ZAN-BPD). All participants also met criteria for a BPD diagnosis according to DSM-5 criteria, confirmed by structured clinical interview. Further exclusion criteria, as documented by the authors of the trial [40], are listed in Supporting Material.

Trial participants were assigned to 13 weeks of either brexpiprazole, or placebo treatment. All participants included in our study completed the p-FBT at the baseline visit, before being allocated to either drug or placebo treatment. A small number of participants (*N* = 26) completed a second p-FBT at one of their further study visits, enabling us to assess drug effects, though this was not our primary interest (see Supporting Material for results).

### Control participants from the general population

Control participants were recruited from the general population in the United Kingdom via the online platform Prolific.co. The experiment and written task instructions were identical for the control and BPD participants. Control participants were matched to the BPD sample for age (±4 years), gender (male, female, non-binary), and educational level (high school diploma/GCSE/A-level, bachelor’s degree, post-graduate degree). We did not explicitly screen these participants for diagnoses of BPD. Nor did we stipulate additional inclusion or exclusion criteria besides the aforementioned matching characteristics. Rather, we addressed the possibility that some control participants might have BPD symptoms by correlating model parameters with self-reported symptom scores.

To increase statistical power, we aimed to recruit two matched control participants for each BPD participant, i.e. 76 control participants. We successfully recruited 75 matched controls. After consenting to take part, and prior to completing the task, control participants met with an experimenter through a video link to ensure that they were able to complete the task in a quiet place, free from distractions or interruptions. One control participant was excluded prior to data analysis since the video-call established that they were in a noisy environment (final *N* = 74 controls).

Procedures for control participants were approved by the University College London Research Ethics Committee (project ID number 20399/001). Participants were compensated for their time at a rate of at least £8.91 per hour. Informed consent was obtained from all participants. All participants had study procedures fully explained to them, and had an opportunity to ask any questions before consenting to take part.

### Probabilistic false belief task (p-FBT)

In the p-FBT, participants observed binary outcomes sampled from a Bernoulli distribution, with underlying probability, *P*^1^. Within the cover story of the task, participants observed whether successive customers arriving at a tourist store on a tropical island purchased either pink umbrellas (1) or yellow sunshades (0) [37]. Periodically, participants were asked to predict *P*, based on recent purchases.

An hypothetical other person, the ‘store manager’, received partial or misleading information about outcomes (Fig. 1a). The probability (*P*) of observing a given outcome, therefore, evolved with differing trajectories for the participant $({P}_{{{\rm {Self}}}})$ and the manager $({P}_{{{\rm {Other}}}})$ (Fig. 1b). Specifically, participants were told that the manager was sometimes with them, and able to see purchases. These ‘Shared’ sampling trials were indicated to the participant as a cartoon face looking through an open door (Fig. 1a). Sometimes, however, the manager was in a back room, and could not see purchases. These ‘Privileged’ sampling trials were indicated as a cartoon face behind a closed door. Finally, participants were told that the manager sometimes observed purchases on a security camera; however, unbeknown to the manager, the footage was out of date, and thereby uninformative about what customers were really buying. These ‘Decoy’ sampling trials were indicated as a cartoon face and an icon showing a computer disc. Each *sampling* trial was presented for 1250 ms, followed by a variable intertrial interval with a fixation cross on screen for 400–600 ms.

Pre-generated sequences of 360 outcomes (purchases) were sampled from two uncorrelated random walks, governing ${P}_{{{\rm {Self}}}}$ and ${P}_{{{\rm {Other}}}}$ (with step sizes of 0.025, bounded between 0 and 1). ‘Privileged’ samples were drawn from ${P}_{{{\rm {Self}}}}$ whilst ‘decoy’ samples were drawn from ${P}_{{{\rm {Other}}}}$. ‘Shared’ samples were drawn randomly from either walk.

After observing a series of purchases (every 4–9 sampling trials), participants were asked to predict the upcoming purchase, in a *probe* trial Specifically, participants were asked to report the probability that the next customer would buy a pink umbrella as opposed to a yellow sunshade, by placing a cursor on a continuous probability scale, either from their own perspective (‘Self’ probe trials; ${P}_{{{\rm {Self}}}}$) or from the perspective of the manager (‘Other’ probe trials; ${P}_{{{\rm {Other}}}}$). In the instructions concerning Other-probe trials, participants were briefed to “pay attention to which sales the manager has and has not seen (including what he has seen on the security footage)”.

Participants were provided with thorough written instructions on the task, including an introductory example, explaining the different trial types and how to use the rating scale. Following this practice phase, participants were asked to rate their understanding of the instructions on a five-point scale, from “Very poor—I do not understand what I am being asked to do” to “Very good—I clearly understand what I am being asked to do”. Participants were asked to repeat the instructions and practice phase if they rated their understanding as poor or very poor. 89% of participants rated their understanding of the instructions as ‘Very good’ or ‘Good’.

Aside from the introductory example, wherein feedback was given, participants received no feedback about their performance. They were briefed that the task was subjectively difficult, and encouraged to do their best. A self-paced break was introduced halfway through the task, which was approximately 15 min in duration.

A laboratory (in-person) version of the task was coded using Matlab (Mathworks, Provo), and visualised using Cogent 2000 (v125) and Cogent Graphics (v1.29). An identical online version was coded using Gorilla.sc.

### Computational model of agent-specific belief updating

We fitted participants’ responses with a simple reinforcement-learning model, wherein beliefs ($B$) about *P* are updated proportionally to a *prediction error* $\left({{\rm {PE}}}\right),$ given by the difference between new observations (1 or 0) and existing predictions. To model agent-specificity, we instantiated parallel learning processes for Self and Other, as follows:

1 $$\begin{array}{c}{B}_{t}^{{{\rm {Self}}}}=\,{B}_{t-1}^{{{\rm {Self}}}}\,+\alpha \left({{{\rm {PE}}}}_{t}^{{{\rm {Self}}}}+{\lambda }^{{{\rm {Other}}}}\cdot {{{\rm {PE}}}}_{t}^{{{\rm {Other}}}}\,\right)+\delta (0.5-{B}_{t-1})\\ {B}_{t}^{{{\rm {Other}}}}=\,{B}_{t-1}^{{{\rm {Other}}}}\,+\alpha ({{{\rm {PE}}}}_{t}^{{{\rm {Other}}}}+{\lambda }^{{{\rm {Self}}}}\cdot {{{\rm {PE}}}}_{t}^{{{\rm {Self}}}}\,)+\delta (0.5-{B}_{t-1})\end{array}$$

Here, ${B}^{{{\rm {Self}}}}$ denotes the subject’s belief, while ${B}^{{{\rm {Other}}}}$ denotes the subject’s representation of the other person’s belief (bounded between 0 and 1). Thus, two belief representations can be separately updated, based on observations available to each agent, and this design feature furnishes agent-specific prediction errors (${{{\rm {PE}}}}^{{{\rm {Self}}}}$ and ${{{\rm {PE}}}}^{{{\rm {Other}}}}$). $0 < \alpha < 1$ is a learning rate parameter, governing sensitivity to new information, while $0 < \delta < 1$ is a memory decay rate, whereby beliefs drift towards chance level. Importantly, updates for Self and Other may not be fully segregated. We capture this effect by invoking ‘leakage’ parameters, ${\lambda }^{{{\rm {Self}}}}$ and ${\lambda }^{{{\rm {Other}}}}$, bounded between -1 and 1, which allow prediction errors pertinent to belief representation of one agent to erroneously update the belief representation of the other agent.

This form of model has previously been found to approximate responses well on the p-FBT [37, 38]. In keeping with previous approaches, ${{{\rm {PE}}}}_{{{\rm {Self}}}}$ was modelled as 0 on Decoy trials, while ${{{\rm {PE}}}}_{{{\rm {Other}}}}$ was modelled as 0 on Privileged trials [37, 38]. Distinct from previous applications, we allowed negative values of ${\lambda }_{{{\rm {Self}}}}$ and ${\lambda }_{{{\rm {Other}}}}$, which allows for anti-correlation in Self- and Other-attributed beliefs (see below).

The task was designed such that, when ${\lambda }^{{{\rm {Self}}}}=0$ and ${\lambda }^{{{\rm {Other}}}}=0$, corresponding to *agent-specific* updating, belief trajectories of Self and Other are uncorrelated (Fig. 1b, c). However, leakage in updates induces a correlation in beliefs across agents; as either ${\lambda }^{{{\rm {Self}}}}$ or ${\lambda }^{{\rm {{Other}}}}$ approaches one, corresponding to *agent-invariant* updating, beliefs for Self and Other converge (Fig. 1b, c). The model also allows for anti-correlation in predictions for Self and Other, which occurs when leakage parameters are less than zero (Fig. 1c). Anti-correlated beliefs imply that, if the subject predicts a given outcome (e.g., pink umbrella), they tend to think the other person will predict the opposite (e.g., yellow sunshade). Leakage parameters thus capture a continuum between self-other mergence on the one hand and self-other differentiation on the other. We hypothesized that participants with BPD would exhibit higher degrees of agent-invariance in belief updates compared with general population controls, manifest as more positive $\lambda$.

Finally, the model allows asymmetric leakage, which occurs if ${\lambda }^{{{\rm {Self}}}}\ne {\lambda }^{{{\rm {Other}}}}$. Here, ${\lambda }^{{{\rm {Self}}}}$ captures how a prediction error experienced by the self generalises to another agent. We refer to such updating as ‘idiocentric’, denoting the fact that, in this instance, one’s own observations generalise to others. Behaviourally, this induces a correlation between estimates of the Other’s belief and outcomes observed by Self (Fig. 1d).

Updating can also be ‘allocentric’, denoting generalisation of prediction error from Other to Self, captured by ${\lambda }^{{{\rm {Other}}}}$. Under allocentric updating, this information influences what the subject comes to believe about the world, inducing a correlation between the subject’s belief and outcomes observed by the Other (Fig. 1e).

### Model fitting procedure

We tested models with the following configurations of leakage parameters: (i) independent ${\lambda }^{{{\rm {Self}}}}$ and ${\lambda }^{{{\rm {Other}}}}$ ($\lambda$ Unrestricted, as shown in Eq. (1)). (ii) ${\lambda }^{{{\rm {Self}}}}={\lambda }^{{{\rm {Other}}}}$ (i.e., symmetric leakage), and iii) a both $\lambda$ parameters set to zero (i.e., a fully agent-specific model). We tested each of these three models with learning rates for Self and Other which were either (i) constrained to be equal ($\alpha ={\alpha }^{{{\rm {Self}}}}$ = ${\alpha }^{{\rm {{Other}}}}$, as shown in Eq. (1) or (ii) could differ ($\alpha \,$Unrestricted). This yielded a set of six models.

Each model yields a point estimate of ${B}^{{{\rm {Self}}}}$ and ${B}^{{{\rm {Other}}}}$ on each trial. These model-derived belief estimates were used to derive the likelihood of a participant’s responses on probe trials. Since participants were asked to estimate ${P}_{{{\rm {Self}}}}$ and ${P}_{{{\rm {Other}}}}$, which are bounded between 0 and 1, we used a Beta distribution for the likelihood, with a mode set to the model-derived point estimate (for details see Supporting Material). For each model, the variance of the Beta distribution was set to a participant-specific parameter, $\tau$, governing the degree of stochasticity in responses. Higher settings of $\tau$ entailed more random responding. For each model, choice temperature ($\tau$) and memory decay ($\delta$) parameters were set to be identical for Self- and Other-updates. Parameters were bounded through sigmoid transformation and fitted in inverse sigmoid space. Both $\alpha$ and *δ* parameters were bounded between 0 and 1; $\tau$ between 0.001 and 0.08 (this upper bound on $\tau$ produces a near-uniform Beta distribution, corresponding to random responding).

For each participant we sought model parameters with maximum posterior probability, given the data. We used fixed Gaussian priors over parameters and assumed a uniform prior over models (see Supporting Material for details of the model fitting procedure). For each model, we calculated the Bayesian model evidence, which favours models that provide a closer fit to the data, whilst penalising more complex models. We first compared models in a fixed-effects approach, summing model evidences across participants. We also examined the frequency of models across participants, in a random-effects approach. For each model we calculated a ‘protected exceedance probability’, a statistic estimating the probability that a given model amongst a set of models is most frequently the best-fitting [41, 42].

To measure parameter recovery for the best fitting model, we fitted the model to data generated for 560 simulated participants. Simulated participants’ parameters were randomly sampled from the observed maximum a posteriori parameter estimates. Each parameter was sampled independently, with replacement. We computed a Pearson correlation coefficient between the generative parameters and fitted parameter estimates.

### Outcome–belief correlations

**Fig. 2:**
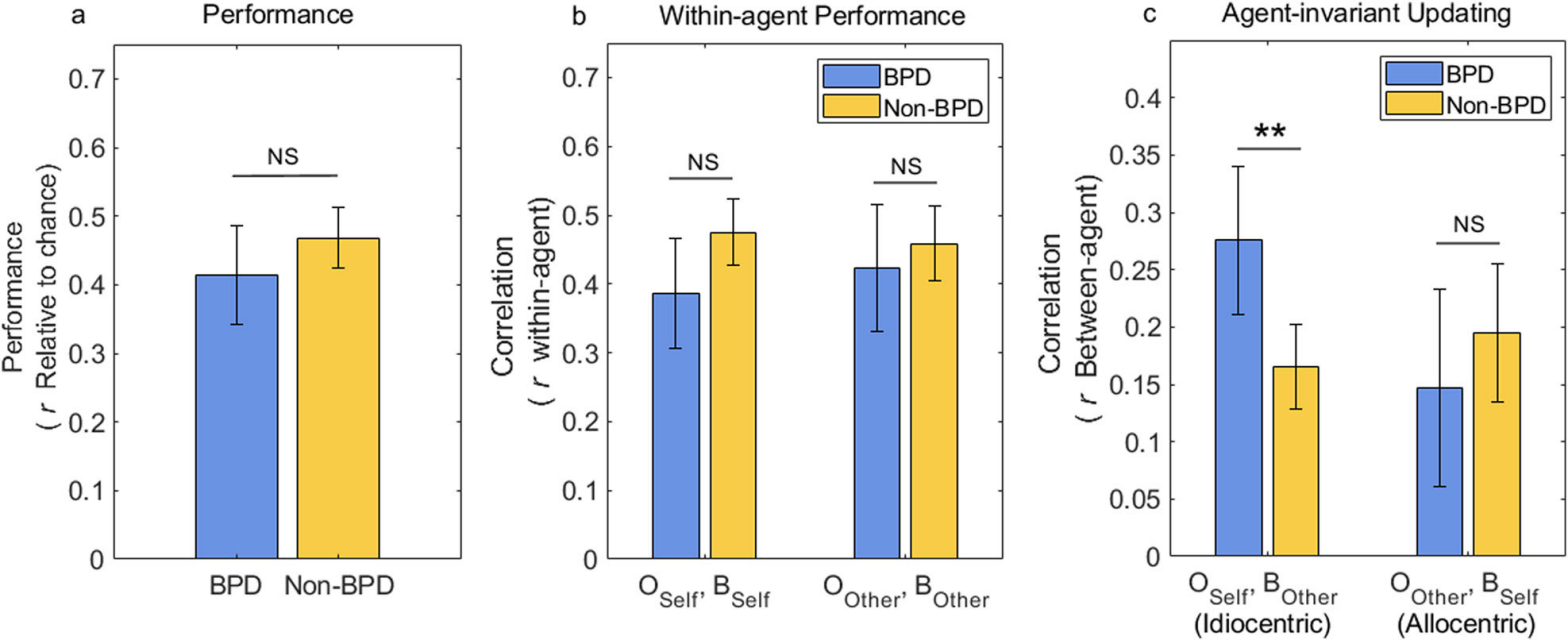
Task performance and outcome-belief correlations. **a** BPD and Non-BPD groups did not differ significantly in performance, expressed as the correlation between participants’ belief estimates and a recency-weighted average of past outcomes (*t*(110) = −1.38, two-tailed *p* = 0.170). Performance is shown relative to that expected under random responding, such that zero indicates chance level. **b** Performance by an agent. Correlations are shown between outcomes pertinent to Self (${O}^{{{\rm {Self}}}}$) and the subject’s reported belief (${B}^{{{\rm {Self}}}}$) on Self probe trials, and between outcomes pertinent to Other (${O}^{{{\rm {Other}}}}$) and belief attributed to the Other (${B}^{{{\rm {Other}}}}$) on Other probe trials. Performance for neither agent differed significantly between BPD and Non-BPD groups (Self: *t*(110) = −1.06, *p* > 0.25; Other: *t*(110) = −0.03, *p* > 0.25). **c** Correlation between outcomes observed by Self (${O}^{{{\rm {Self}}}}$) and belief attributed to the Other (${B}^{{{\rm {Other}}}}$), a measure of idiocentric updating, was significantly greater in the BPD group than in the Non-BPD group (*t*(110) = 3.12, *p* = 0.002). Correlation between outcomes observed by the Other (${O}^{{{\rm {Other}}}}$) and the subject’s reported belief (${B}^{{{\rm {Self}}}}$), a measure of allocentric updating, did not differ significantly between groups (*t*(110) = −0.86, *p* > 0.25). Error bars show 95% confidence intervals. **p < 0.01, NS: *p* > 0.05.

We derived ‘model-free’ measures of belief updating on the task. Firstly, we measured task performance by correlating a participant’s reported beliefs about ${P}_{{{\rm {Self}}}}$ and ${P}_{{{\rm {Other}}}}$ on probe trials with an exponential recency-weighted average, *O*, of past outcomes for Self and Other respectively (see Supporting Material for details). We used a recency-weighted average, rather than the true generating *P*, so as to account for sampling error in the observed outcomes. For each participant’s trial sequence, we generated a null distribution for this outcome–belief correlation, by generating 1000 simulated sets of random responses. We expressed performance relative to that expected under this null distribution, such that a score of zero indicates chance-level responding (shown in Fig. 2a). Secondly, we examined performance in each participant group for Self and Other probe trials separately, i.e., *within-agent* outcome-belief correlations.

Finally, as a behavioural measure of agent-invariant updating, we examined *across-agent* outcome-belief correlations using the same method. Correlation between estimates of the Other’s belief (${B}^{{{\rm {Other}}}}$) and a weighted-average of outcomes observed by Self (${O}^{{{\rm {Self}}}}$) (on Privileged and Shared trials), measures idiocentric updating, while the correlation between the subject’s reported belief (${B}^{{{\rm {Self}}}}$) and outcomes observed by the Other (${O}^{{{\rm {Other}}}}$) (on Decoy and Shared trials) measures allocentric updating.

### Symptom questionnaires

All participants completed self-reported questionnaires to assess BPD severity, of which our primary outcome of interest was the Borderline Symptom List [39] (BSL-95). Participants also completed the Zanarini Self-Report scale [43] (Zan-SR). Impulsivity was assessed using the Barratt Impulsiveness Scale (BIS-11) [44]. In addition, we used standardised questionnaires to assess affective symptoms; for BPD participants, clinician-rated measures were administered, namely the Hamilton Depression Rating Scale [45] (HAM-D), and Young Mania Rating Scale [46] (YMRS), while control participants completed self-report scales, namely the Inventory of Depressive Symptomatology [47] (IDS), and Altman Mania Rating Scale [48] (AMRS).

To examine relationships between BPD symptoms and agent-invariant updating, we used univariate simple linear regression, with ${\lambda }^{{{\rm {Self}}}}\,$(or ${\lambda }^{{{\rm {Other}}}}$) as the dependent variable, and BSL-95 score as the independent variable. To account for possible between-group differences in the effect of symptom scores, we also included a group (BPD vs. Control) by symptom score (BSL-95) interaction term. In a second regression analysis, we also included symptoms of impulsivity, depression and mania, and demographic factors (age, gender, ethnicity, educational level and employment) as covariates. Since depression and mania scales differed between BPD and Control groups, we expressed total scores as percentages of the maximum score on each scale. All statistical tests reported were two-sided.

## Results

### Group characteristics

Supporting Table S1 shows demographics and symptom scores for the BPD and control groups. Additional characteristics of the BPD sample, such as co-morbid diagnoses, drug and alcohol use, or concomitant medications, are provided in Supporting Table S2.

### Outcome-belief correlations

Both BPD and control groups performed above chance on average (BPD: mean = 0.41, 95% CI [0.34 0.49] one-sample *t*(37) = 11.6, *p* < 0.001; Non-BPD: mean = 0.47 95% CI [0.42 0.51], t(73) = 21.4, *p* < 0.001), and performance did not differ significantly between the two groups (Fig. 2a; 95% CI of performance difference [−0.13 0.02], two-sample *t*(110) = −1.38, two-tailed *p* = 0.170). Performance was above chance in 107 out of 112 participants. In 95 out of 112 participants, performance was above the 95th percentile of a null distribution generated by random responding. In pairwise comparisons, performance for neither agent differed significantly between BPD and control groups, as shown by *within-agent* outcome-belief correlations. (Fig. 2b; *Self*: 95% CI of difference [−0.14 0.04], *t*(110) = −1.06, *p* > 0.25; Other: 95% CI [−0.10 0.10], *t*(110) = −0.03, *p* > 0.25). Thus, we found no evidence that participants were systemically ignoring outcomes pertinent to either agent.

As a behavioural test of agent-invariant updating, we next examined *between-agent* outcome-belief correlations. Pairwise tests revealed a significantly greater idiocentric updating (${O}^{{{\rm {Self}}}},{B}^{{{\rm {Other}}}}$ correlation) in the BPD group compared to the control group (95% CI of difference [0.04 0.18]; *t*(110) = 3.12, *p* = 0.002), with no significant difference in allocentric updating (95% CI of difference in ${O}^{{{\rm {Other}}}},{B}^{{{\rm {Self}}}}$ correlation [−0.15 0.06]; *t*(110) = −0.86, *p* > 0.25). These results indicate that BPD participants tended to use their own observations to estimate the Other’s belief to a greater extent than control participants from the general population.

### Model comparison

**Fig. 3:**
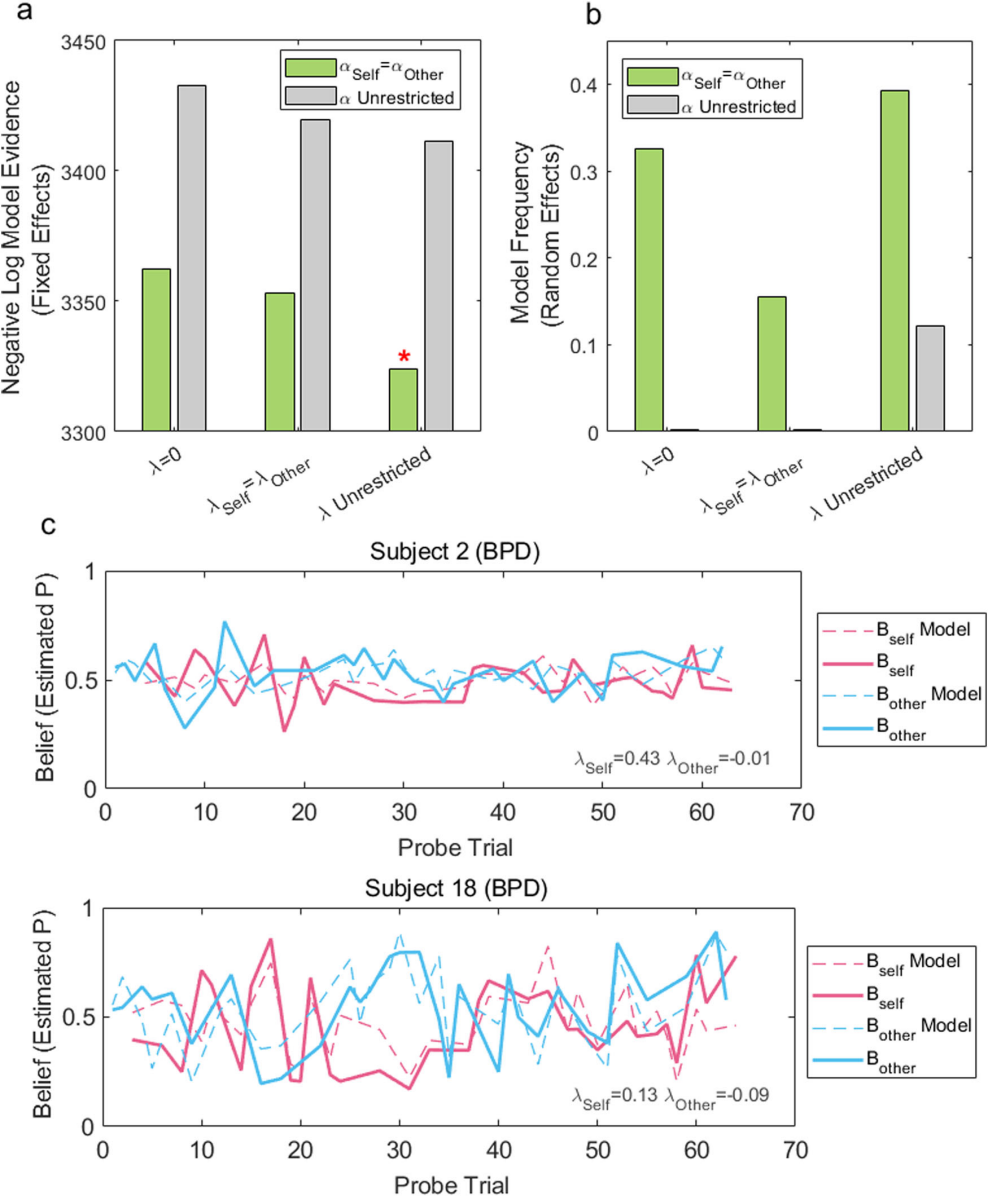
Model comparison and model fits. **a** Negative log model evidence, summed across all participants (*N* = 112), for different model configurations. Lower values indicate a better model fit. $\lambda =0$ entails fully agent-specific learning; ${\lambda }^{{{\rm {Self}}}}$ = ${\lambda }^{{{\rm {Other}}}}$ denotes that idiocentric and allocentric updating are constrained to be equal; $\lambda$ Unrestricted denotes that idiocentric (${\lambda }^{{{\rm {Self}}}}$) and allocentric (${\lambda }^{{{\rm {Other}}}}$) updating can differ. Learning rates for Self and Other were either constrained to be equal (${\alpha }^{{{\rm {Self}}}}$ = ${\alpha }^{{{\rm {Other}}}}$) or could differ ($\alpha \,$Unrestricted). A model with unrestricted $\lambda$, and equal learning rates (as shown in Eq. (1)) provided the best fit, indicated by an asterisk. **b** Frequency of best-fitting models across participants. A model with unrestricted $\lambda$, and equal learning rates was the most frequent model, with a protected exceedance probability of 0.802. **c** Reported beliefs and model predictions from the best fitting model are shown for two sample participants. Self and Other probe trials are interleaved in the order presented to participants.

We went on to fit learning models to participants’ belief estimates for Self and Other (Fig. 3). In essence, this involved minimising differences between belief estimates derived from each model, and those reported by participants. Models with independent ${\lambda }^{{{\rm {Self}}}}$ and ${\lambda }^{{{\rm {Other}}}}$ ($\lambda$ Unrestricted) outperformed those in which leakage was constrained to be symmetric $({\lambda }^{{{\rm {Self}}}}={\lambda }^{{{\rm {Other}}}})$, or no leakage $(\lambda =0)$ (Fig. 3a, b). Also, models in which learning rates for Self and Other were constrained to be equal outperformed those in which learning rates could differ by agent.

In fixed-effects model comparison, a model with Unrestricted $\lambda$, and equal learning rates for Self and Other (as shown in Eq. (1)) provided the best overall fit (fixed-effect Bayes factor for $[{\lambda }^{{{\rm {Self}}}}\ne {\lambda }^{{{\rm {Other}}}},\,{\alpha }^{{{\rm {Self}}}}={\alpha }^{{{\rm {Other}}}}]$ vs. $[{\lambda }^{{{\rm {Self}}}}={\lambda }^{{{\rm {Other}}}},\,{\alpha }^{{{\rm {Self}}}}={\alpha }^{{{\rm {Other}}}}]$ = 29.5). This model was also the most frequent in a random-effects model comparison (best-fitting model in 39% of participants, protected exceedance probability 0.802; Fig. 3b). As shown in Fig. 3c, the model estimated participants’ choices well.

Notably, although a model with independent ${\lambda }^{{{\rm {Self}}}}$ and ${\lambda }^{{{\rm {Other}}}}$ provided the best overall fit, some participants’ responses were more parsimoniously captured with either a single leakage parameter or no leakage. Since the models are nested, these individual differences ought to be reflected in the fitted parameters of the more general model. This was indeed the case. For example, participants for whom a model with no leakage was favoured had ${\lambda }^{{{\rm {Self}}}}$ estimates closer to zero (mean ${\lambda }^{{{\rm {Self}}}}$ = 0.0003) than those for whom models with leakage were favoured (mean ${\lambda }^{{{\rm {Self}}}}$ = 0.07). When comparing parameters between BPD and control groups, we therefore used the more general model.

### Parameter recovery

Correlations amongst the fitted parameters in the participant sample are shown in Supporting Material in Fig. S1; parameter recovery for the best-fitting model is shown in Fig. S2. ${\lambda }^{{{\rm {Self}}}}$ and ${\lambda }^{{{\rm {Other}}}}$ were negatively correlated across participants (Pearson *r* = −0.19). Notably, however, in parameter recovery these two parameters were separately recoverable, i.e., fitted ${\lambda }^{{{\rm {Self}}}}$ was uncorrelated with generative ${\lambda }^{{{\rm {Other}}}}$. Leakage parameters were also recovered separately from memory decay ($\delta )$, learning rate ($\alpha$) or choice noise ($\tau$) parameters (Fig. S1a).

Accuracy for the recovery of$\,{\lambda }^{{Self}}$ and ${\lambda }^{{Other}}$ was relatively modest (*r* = 0.63 and 0.53 respectively). This was partly attributable to including high settings of choice noise in the recovery analysis. When the analysis was restricted to the lowest quartile of generative $\tau$, recoverability of$\,{\lambda }^{{{\rm {Self}}}}$ and ${\lambda }^{{{\rm {Other}}}}$ improved (*r* = 0.85 and 0.82, respectively; Fig. S2b).

Taken together, these findings indicate that our task design can robustly distinguish directions of leakage. This conclusion, from a model-based analysis, is also consistent with our ‘model-free’ analyses, which show how estimates of leakage can be derived from first principles, based on between-agent outcome–belief correlations. It appears however that leakage is less reliably estimable at higher settings of choice noise.

### Group comparison of agent-invariant updating

**Fig. 4:**
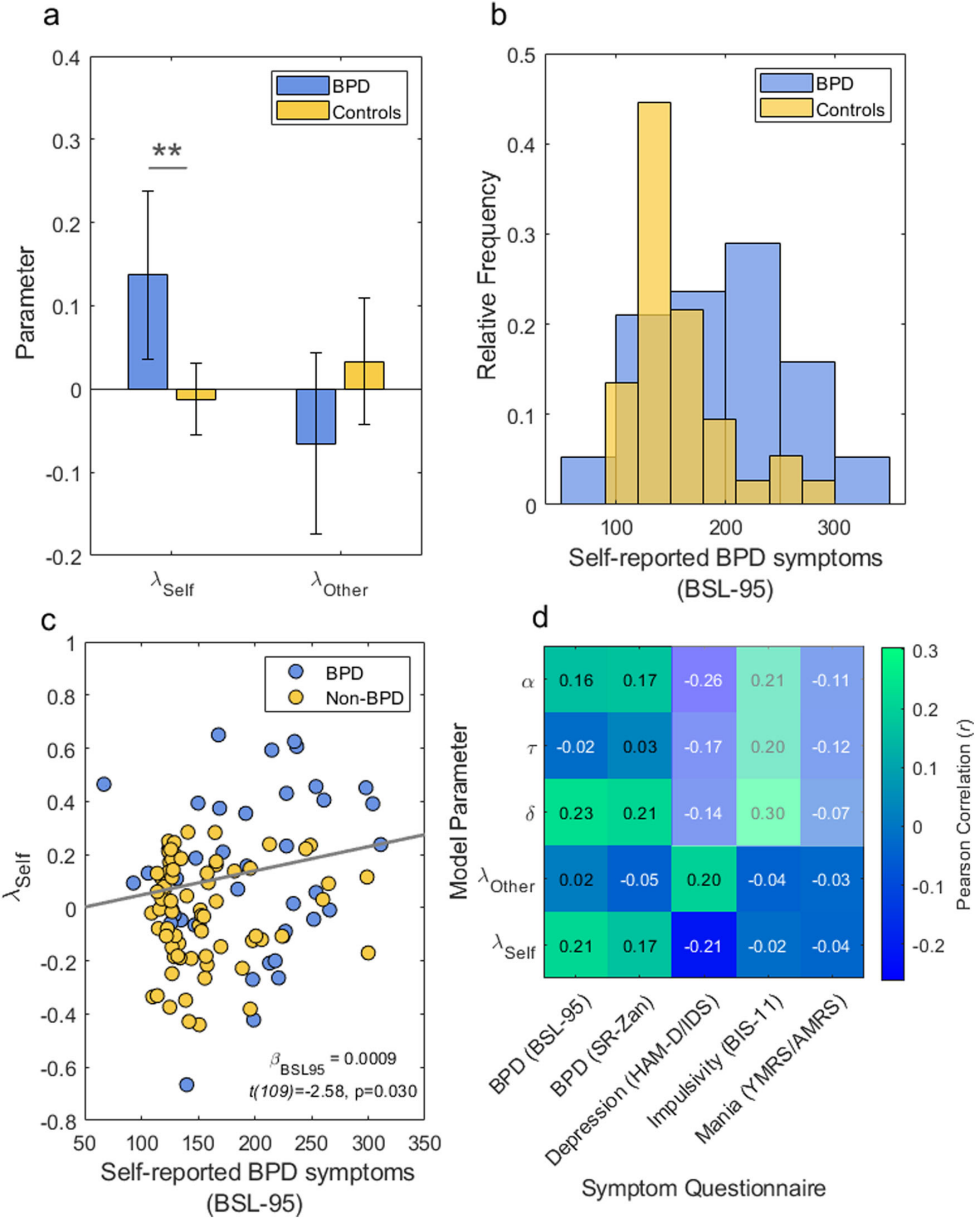
BPD is associated with greater idiocentric updating. **a** Mean estimates of leakage parameters in BPD and Non-BPD groups. Idiocentric updating, captured by ${\lambda }^{{Self}}$, was higher amongst BPD participants than amongst Non-BPD controls (two-sample, *t*(110) = 3.19, *p* = 0.002; Cohen’s *d* = 0.64); there was no significant difference in ${\lambda }^{{{\rm {Other}}}}$ between the two groups (*t*(110) = −1.50, *p* = 0.137). **b** Distribution of self-reported BPD symptoms (BSL-95 scores) across BPD and control groups. **c** Idiocentric updating correlated with BPD symptoms across all participants (solid grey line indicates the main effect of BSL-95 scores). **d** Pearson correlations between all model parameters and symptom scores across all participants (*N* = 112). The translucent white box indicates combinations of symptom scores and parameters that were not of prior interest. In multiple regression on ${\lambda }^{{{\rm {Self}}}}$ with BPD symptoms (BSL-95), depression, mania, impulsivity, age, gender, ethnicity, education and employment status as predictor variables, BPD symptoms emerged as the only significant predictor (${\beta }_{{{\rm {BSL}}}-95}=0.0014$; *t*(82) = 2.73, *p* = 0.0078). BSL-95: 95 item Borderline Symptom List; SR-Zan: Self-report Zanarini scale; HAM-D: Hamilton Depression Rating Scale; IDS: Inventory of Depressive Symptomatology; BIS-11: Barratt Impulsiveness Scale; YMRS: Young Mania Rating Scale; AMRS: Altman Mania Rating Scale.

We found that ${\lambda }^{{{\rm {Self}}}}$ was significantly higher amongst BPD participants than amongst general population controls (95% CI of difference [0.06 0.24]; two-sample, *t*(110) = 3.19, *p* = 0.002; Cohen’s *d* = 0.64), with no significant difference in ${\lambda }^{{{\rm {Other}}}}$ between the two groups (95% CI [−0.23 0.03]; *t*(110) = −1.50, *p* = 0.137). In essence, we found a significant effect of BPD diagnosis on leakage from Self-relevant learning signals to estimates of the Other’s belief, captured by ${\lambda }^{{{\rm {Self}}}}$(Fig. 4a). Mean ${\lambda }^{{{\rm {Self}}}}$ was greater than zero in BPD participants (mean 0.14; 95% CI [0.04 0.24], one-sample *t*(37) = 2.75, *p* = 0.009), and not significantly different from zero in control participants (mean −0.01, 95% CI [−0.06 0.03], *t*(73) = −0.55, *p* > 0.25). Thus, BPD participants generalised their own belief updates to others (positive ${\lambda }^{{{\rm {Self}}}}$), while control participants showed no significant degree of generalisation across agents (see also Fig. 4a). This finding suggests that control participants, relative to BPD participants, were more capable in mentalising differences between Self and Other within the task.

### Correlation between agent-invariant updating and BPD symptoms

We next tested for a relationship between agent-invariant updating and BPD symptom severity (measured by the BSL-95) across participants. Notably, although mean BSL-95 scores were significantly higher amongst BPD participants than amongst controls from the general population (mean BPD = 198.1, mean controls = 153.5, *t*(112) = 4.5, *p* < 0.001), substantial variability was observed in both groups (Fig. 4b). Perhaps surprisingly, some of the lowest scores were observed amongst the BPD group. One explanation for this is the possibility this reflects measurement error (variable responding) in the BPD group, since all BPD participants scored at least nine points on the clinician-rated Zanarini Rating Scale, and these clinician-rated scores were positively correlated with BSL-95 scores (*r* = 0.45, *p* = 0.005).

${\lambda }^{{{\rm {Self}}}}$ showed a significant positive relationship with BPD symptoms (${\beta }_{{{\rm {BSL}}}95}$ = 0.0009*,t*(109) = 2.20, *p* = 0.0300; Fig. 4b). Thus, idiocentric updating was positively related to BPD symptoms across participant groups. We also observed a significant interaction between BPD symptoms and participant group, with a larger effect of symptoms amongst the BPD group than amongst the control group (${\beta }_{{{\rm {BSL}}}95{x\; {\rm {group}}}}$ = −0.0007*, t*(109) = −2.58, *p* = 0.011). A possible explanation for this interaction is that more substantial impairments in mentalising (corresponding to higher settings of ${\lambda }^{{{\rm {Self}}}}$) translate into clinically meaningful symptoms, while subtle mentalising difficulties may remain asymptomatic.

A relationship between ${\lambda }^{{{\rm {Self}}}}$ and BPD symptoms remained significant after controlling statistically for symptoms of depression, mania, impulsivity, age, gender, ethnicity, education and employment status, by including these as covariates (${\beta }_{{{\rm {BSL}}}-95}=0.0014$; *t*(82) = 2.73, *p* = 0.0078; see Supporting Table S3 for full regression results). In this analysis, no other symptom scores were significantly related to ${\lambda }^{{{\rm {Self}}}}$.

In a separate regression, we found no significant relationship between ${\lambda }^{{{\rm {Other}}}}$ and BPD symptoms (${\beta }_{{{\rm {BSL}}}-95}=0.0001,\,$*t*(109) = 0.25, *p* > 0.25). This negative result is consistent with our finding, in behavioural analyses, of no significant difference between BPD and control groups in allocentric updating, as measured by an ${O}^{{{\rm {Other}}}},{B}^{{{\rm {Self}}}}$ correlation. Fixed effect pairwise correlations between all model parameters and symptom scores are shown in Fig. 4c. We also observed a positive correlation between BPD symptoms and the memory decay parameter, $\delta$. Notably however $\delta$ was separately recoverable from ${\lambda }^{{{\rm {Self}}}}$ in a parameter recovery analysis (Fig. S1).

### Sensitivity analyses

To ensure that the reported effects are not solely attributable to participants whose task performance did not differ from chance, we repeated the key analyses, excluding participants (*N* = 17) for whom we could not reject a null hypothesis of chance responding. Amongst the remaining 95 participants, ${\lambda }^{{{\rm {Self}}}}$ remained significantly higher amongst BPD participants than amongst general population controls (95% CI of difference [0.04 0.24]; two-sample, *t*(93) = 2.78, *p* = 0.007; Cohen’s *d* = 0.55). Furthermore, a relationship between ${\lambda }^{{{\rm {Self}}}}$ and BPD symptoms remained significant, even when controlling statistically for demographic factors and other symptom scores (${\beta }_{{{\rm {BSL}}}-95}=0.001,\,$95% CI [0.0002 0.002]; *t*(67) = 2.34, *p* = 0.022).

## Discussion

In this study we formalise self-other mergence in terms of generalisation, or ‘leakage’, across agent-representations during learning. Under *idiocentric* updating, one’s own prediction errors generalise to others, implying that a belief that what is surprising for the self is also surprising for others. By contrast, under *allocentric* updating, others’ prediction errors are used to update one’s own belief about the world, implying that others’ surprise is treated as a self-relevant learning signal. To revisit our introductory example, a theatregoer with extreme idiocentric updating would expect Romeo to share their knowledge that Juliet was feigning her death. By contrast, a theatregoer with allocentric updating, upon seeing Romeo’s response, might believe that Juliet was, in fact, dead. Either form of reasoning would render the events of the play highly confusing.

By analysing responses on a probabilistic false belief task (p-FBT) [37, 38], we show that participants with diagnoses of Borderline Personality Disorder (*N* = 38) exhibited higher degrees of idiocentric updating compared with a group of control participants sampled from the general population (*N* = 74). That is, people with BPD tended to generalise their own learning to others. Furthermore, the degree of idiocentric updating correlated with self-reported BPD symptoms across participants, even when controlling statistically for demographic factors, impulsivity, and mood disorder.

### Clinical considerations and potential application of the p-FBT

Clinically, mentalising in BPD deteriorates under conditions of heightened affect, for example following the withdrawal of an attachment figure, creating a vicious cycle where emotional and relational instability potentiate each other [49, 50]. A pertinent question therefore concerns whether idiocentric updating, as measured by the p-FBT, varies dynamically as a function of affective or relational disturbance. Studies using two-person economic games to quantify mentalising support this idea. For instance, computational analyses of such games suggest that representations of the other player’s strategy made by BPD participants tend to collapse when cooperation breaks down [51–53].

Agent-specific updating also suggests a novel outcome-measure for relational psychotherapy, the goal of which is to help individuals form more veridical representations of self and other. This finds support in evidence agent-specific neural representations are modifiable through training [38], and that, in non-human primates, neurons organise over time into agent-specific units for simulating the decisions of others [54]. Notably, by contrast to existing measures of theory-of-mind, such as conventional false-belief tasks [24, 29] or the *faux pas* test [55], the p-FBT entails serial belief updates, with changing underlying probabilities. This renders the task suitable for repeated measurement in a clinical context since it is not possible for the subject to simply learn (or ‘second-guess’) the correct answers.

### Mentalising in BPD: a general deficit in meta-representation?

Notably, the p-FBT differs from many existing paradigms used to investigate mentalising, which examine inference about another person’s feelings and/or intentions. These studies find that, relative to non-BPD controls, people with BPD tend to describe others’ mental states in ways that are either lacking in nuance [56–58] or excessively precise [59–61]. Also, BPD participants have difficulty tracking changes in others’ reliability [62, 63], and in re-establishing cooperation following ruptures of trust [51, 52], while also being slow to revise negative appraisals of others [64, 65]. This echoes the developmental histories of people with BPD, where experiences of neglect and abuse are more frequent than in the general population [66, 67].

By contrast to the aforementioned studies, in the p-FBT, the participant receives no information about the other person’s actions or expressions. Indeed, the p-FBT as implemented here is ‘social’ only in its framing, and requires no inference about strictly interpersonal statistics (such as trustworthiness or generosity). Thus, the behavioural effect we observe is unlikely to depend on a participant’s inference regarding the other’s social characteristics.

Our finding that BPD participants show disrupted mentalising on the p-FBT might therefore be taken to indicate a general difficulty in representing and updating multiple points of view, known as ‘meta-representation’ [68]. This domain-general impairment may, in turn, give rise to more strictly interpersonal aspects of BPD. Imagine, for example, that an employee of a company is paid proportional to the company’s performance; the employee’s impression of business performance could differ from that of their manager. Difficulty representing this difference in perspective could encourage false social inference; for instance, a subject who believed the business was performing well might, following a decrease in their wages, conclude that the manager was deliberately exploiting them by cutting their pay. Such disrupted inferential processes may underpin heightened interpersonal sensitivity observed in BPD.

Difficulty adopting alternative perspectives might also entail challenges in imagining *oneself* in future situations. Indeed, computations necessary for understanding others’ mental states are known to overlap with those involved in representing one’s own [68–70]. Thus, impaired perspective-taking may generate impulsive, as well as interpersonal, features of the BPD syndrome.

A possibility that mentalising deficits observed in BPD are not specific to social cognition begs the question of to what extent the findings reported here are specific to BPD symptomatology. Deficits in theory-of-mind are well documented in other mental disorders, including autistic spectrum disorder (ASD) [71] and schizophrenia [72], whose clinical features partly differ from BPD. Echoing the considerations above, it has been proposed that theory-of-mind deficits in autism lie downstream of a more general impairment in integrating sensory data to form abstract conceptual representations [73]. Social impairments are thought to ensue because reasoning in social situations relies heavily on prior concepts [74]. It appears possible that a similar deficit in information integration might also underpin BPD. This is consistent with a recent view of BPD as a social communication disorder [75], and with conceptual parallels between the symptoms of BPD and ASD [76]. Such a proposal does not obviate the role of childhood trauma in the aetiology of BPD. Rather, a neurodevelopmental predisposition towards social communication difficulties would be expected to interact with the childhood environment to determine the expression of BPD in adulthood.

### Limitations of the current study

Despite the advantages outlined above, the current study has limitations. Firstly, we could reject a null hypothesis of random responding in only 85% of participants. This relatively high rate of random responding does not impact our conclusions, since the key findings remained unchanged whether or not we included random responders in the analysis. Nevertheless, making the task easier (for instance, by increasing stimulus presentation time) would reduce the rates of unusable data. Secondly, a potential limitation of the study itself is that, since data collection was interrupted by the Covid-19 pandemic, a small number of BPD participants (9 out of 38), and all control participants, completed the study online. Mitigating this concern, participants completing the study online met with an experimenter through a video call, to ensure controlled experimental conditions.

### Summary

In summary, our findings are in keeping with a clinical notion of ‘psychic equivalence’ in BPD, wherein mental states are equated with external reality [2, 18, 19]. Our model suggests a candidate computational process underpinning ‘psychic equivalence’, namely an over-generalisation of self-relevant learning signals. Following existing psychological theories, it appears plausible that the resulting fusion of self- and other-representations contributes to the relational and affective instability observed in individuals with BPD [10–12, 14, 22, 23]. Future work might focus on evaluating the computational model described here as a transdiagnostic measure of mentalising ability in clinical settings.

## Supplementary information

Supporting Material Online

Supporting Figure 1

Supporting Figure 2

## Data Availability

The patient data that support the findings of this study are available from JEG upon reasonable request. Patient data are not publicly available due to confidentiality issues. Behavioural data from control participants and computer codes necessary to fit the learning models described here are publicly available at http://github.com/gilesstory/FalseBeliefTask.

## Footnotes

1 A Bernoulli distribution is the probability distribution of a random variable which takes the value 1 with probability *P* and the value 0 with probability 1−*P*.
