# Supplementary material for: A computational signature of self-other mergence in Borderline Personality Disorder
Source: Transl Psychiatry. 2024 Nov 19;14:473. doi: 10.1038/s41398-024-03170-w (PMC11576885; doi:10.1038/s41398-024-03170-w)
Supplement: Supplementary file 1 — Supporting Material Online [file 41398_2024_3170_MOESM1_ESM.docx]

**Supporting Material Online**

A computational signature of self-other mergence in Borderline Personality Disorder

Giles W. Story^1,2^, Sam Ereira^2^, Stephanie Valle^3^, Sam R. Chamberlain^4,5^, Jon E. Grant^3^, Raymond J. Dolan^2^

1. Division of Psychiatry, University College London, UK

2. Max Planck-UCL Centre for Computational Psychiatry and Ageing Research, University College London, UK

3. Department of Psychiatry and Behavioral Neuroscience, University of Chicago, Illinois, USA

4. Department of Psychiatry, Faculty of Medicine, University of Southampton, UK

5. Southern Health NHS Foundation Trust, Southampton, UK

**Supporting Materials and Methods**

**Clinical trial exclusion criteria**

Exclusion criteria, as documented by the authors of the clinical trial from which BPD participants were recruited^61^, were as follows: “unstable medical illness; schizophrenia or bipolar disorder; an active substance use disorder; current pregnancy or lactation, or inadequate contraception in women of childbearing potential; a suicide attempt within the six months before the baseline visit or significant risk of suicide; illicit substance use based on urine toxicology screening (excluding marijuana); initiation of psychological interventions within three months of screening; use of any new psychotropic medication started within the past three months before study initiation; previous treatment with brexpiprazole; and cognitive impairment that might interfere with the capacity to understand and self-administer medication or provide written informed consent” (p59).

**Bayesian model fitting procedure**

For each participant we sought model parameters with maximum posterior probability, given the data. The goal here is to maximise the posterior probability $P\left( \theta| x,M \right)$, where $\theta$ denotes a vector of model parameters, $x$, a vector of observed responses, and $M$ the model under test.

$$P\left( \theta| x,M \right) \sim P\left( \theta\right)P(x|\theta,M)$$

(S1)

Maximising this posterior is equivalent to maximizing the numerator of Bayes' theorem (Eq. S1), since the denominator, $P(x)$, is constant with respect to θ. The likelihood function, $P(x|\theta,M)$, was given by a Beta distribution. Beta distributions are conventionally parameterised with two concentration parameters, $\alpha$ and $\beta$ (note this is a different usage of $\alpha$ from that denoting the learning rate), as follows:

$$P\left( x| \alpha,\beta\right)=\frac{x^{(\alpha-1)}{(1-x)}^{(\beta-1)}}{Beta(\alpha,\beta)}$$

(S2)

where $x$ is an observed probability estimate, and $P\left( x| \alpha,\beta\right)$is the likelihood of that estimate given the concentration parameters of the Beta distribution. The Beta function on the denominator is a normalisation constant. The mode and variance of the Beta distribution can be expressed in terms of the concentration parameters, $\alpha$ and $\beta$, as follows:

$$\frac{\alpha-1}{\alpha+\beta-2}=Mode$$

(S3)

$$\frac{\alpha\beta}{\left( \alpha+\beta\right)^{2}(\alpha+\beta+1)}=Variance$$

(S4)

We set the mode of the Beta distribution equal to the model-derived belief on each probe trial ($B_{t}^{Self}$ on self probe trials and $B_{t}^{Other}$ on other probe trials). We set the variance equal to the participant-specific temperature parameter, $\tau$ (see ^37^). Given these parameters, we solved equations S3 and S4 simultaneously to obtain the concentration parameters, and thus the Beta distribution. The likelihood of a subject’s choice on that trial was obtained using Equation S2. For each subject we summed log likelihoods across trials to obtain the joint likelihood of all choices, conditioned on the current parameter estimates.

The same set of Gaussian priors, $P\left( \theta\right),$ over model parameters was used for all participants. The prior mean for each parameter was set to the median of the upper and lower bounds (inverse sigmoid transformed). The prior standard deviation was set to a quarter of the distance between upper and lower bounds for each parameter (in inverse sigmoid space). This broad Gaussian prior regularised the inference by penalising extreme parameter estimates. Parameters were bounded using a logistic sigmoid transformation. Model functions were written using custom computer code in Matlab (Mathworks, Provo). The maximum *a posteriori* parameters were sought by minimising the negative log of the joint density of prior and likelihood (as shown in Eq. S1) using a built-in unconstrained optimisation algorithm (fminunc).

Bayesian model evidence, $P\left( x | M \right)$, was calculated for each model by marginalising the joint probability, $P\left( x, \theta| M \right)$, with respect to the parameters, $\theta$. The exact calculation of $P\left( x | M \right)$ is given by:

$$P\left( x | M \right)=\int P\left( \theta\right)P\left( x | \theta,M \right) d\theta$$

(S5)

We approximated this integral numerically, by randomly sampling parameters from the prior distribution, such that:

$$P\left( x | M \right)\approx\frac{1}{K} \sum_{k=1}^{K} P\left( x | \theta_{k},M \right)$$

(S6)

Where $\theta_{k}$ is a sampled set of parameters for a given model, and *K* is the total number of samples, set to 2000. We assumed a uniform prior over models.

We used the model evidences to compare models using a random-effects approach, calculating the exceedance probability of each model. This statistic estimates the probability that a given model amongst a set of models is most frequently the best-fitting model across participants.

To generate the plots shown in Fig. 1a and 1b, we simulated beliefs from a model with $\alpha=0.1$, $\tau=0.04$ and $\delta=0$, at varying leakage parameters. To measure parameter recovery for the best fitting model (as shown in Equation 1), we fitted the model to simulated data, generated for 560 simulated participants. Simulated participants’ parameters were sampled from a Gaussian distribution with the same mean and variance as that of the observed maximum *a posteriori* parameter estimates. We computed a Pearson correlation coefficient between the generative parameters and fitted parameter estimates.

**Outcome-belief correlations**

We measured task performance by correlating a participant’s estimates of *P* on probe trials with an exponential recency-weighted average, *O*, of past outcomes, given by:

$$O_{t}=\sum_{\tau=1}^{t-1} o_{\tau}\omega_{\tau}$$

(S7)

Where $\boldsymbol{\omega}=[\omega_{1},\omega_{2}\ldots\omega_{t-1}]$ are a set of normalised weights, given by:

$$\omega_{\tau}=\frac{\gamma^{t-\tau}}{\sum_{\tau=1}^{t-1} \gamma^{t-\tau}}$$

(S8)

Where $0<\gamma<1$ is a decay rate. We used a recency-weighted average, rather than the true generating *P*, so as to account for sampling error in the observed outcomes. On Other-probe trials we averaged recent outcomes observed by the Other (on Decoy and Shared sampling trials). On Self-probe trials, we averaged recent outcomes observed by the Self (on Privileged and Shared sampling trials).

For each participant’s trial sequence, we generated a null distribution for this outcome-belief correlation, by generating 1000 simulated sets of random responses. We expressed performance relative to that expected under this null distribution, such that a score of zero indicates chance-level responding (shown in Fig. 2a). To test for significantly above-chance responding we also expressed performance relative to the 95^th^ percentile of the null distribution; here a score greater than zero indicates significantly above-chance responding at a within-subject level. We calculated these relative performance measures at a range of exponential decay rates in the weighted average ($\gamma=[0.1$, 0.3, 0.5, 0.7, 0.9]) and averaged the estimates. We also examined performance in each participant group for Self and Other probe trials separately, i.e., *within-agent* outcome-belief correlations.

Finally, as a behavioural measure of agent-invariant updating, we examined *across-agent* outcome-belief correlations using the same method. Correlation between estimates of the Other’s belief ($B^{Other}$) and a weighted-average of outcomes observed by Self ($O^{Self}$) (on Privileged and Shared trials), measures idiocentric updating, while correlation between the subject’s reported belief ($B^{Self}$) and outcomes observed by the Other ($O^{Other}$) (on Decoy and Shared trials) measures allocentric updating.

**Supporting Results**

**Drug effects on agent-specific updating**

A subset of BPD participants (*N*=26) also completed a second p-FBT, allowing us to examine drug effects, though this was not our primary interest. Amongst BPD participants who completed the p-FBT before and after randomisation to either brexpiprazole or placebo (*N*=26), we found no significant effect of brexpiprazole on idiocentric updating ($\lambda^{Self}$) after randomisation, controlling for baseline $\lambda^{Self}$ (*F*(3,23)=0.06, *p*>0.25). We note however that this analysis was exploratory, and likely underpowered to detect subtle effects.

**Supporting Figures and Tables**

**Fig. S1 Fitted parameter correlations**

Correlation matrix of maximum a posteriori parameter estimates for the best-fitting model. Each cell shows a Pearson correlation coefficient between the parameter estimates. Correlations on the diagonal amount to those between a parameter and itself.

**Fig. S2 Parameter recovery**

Parameter recovery for the best fitting model. We fitted the model to simulated data generated for 560 simulated participants. Each cell shows a Pearson correlation coefficient between the generative and recovered parameter estimates. **a** Simulated participants’ parameters were sampled independently, with replacement, from the maximum *a posteriori* parameter estimates estimated from the observed participant data. **b** Generative choice noise, $\tau$, was sampled from the lowest quartile of the observed parameter distribution. This improved the accuracy of parameter recovery.

**Table S1. Demographics and Symptom Scores in BPD and Control Groups**

| Variable | BPD | General Population Controls | *p*-value |
| --- | --- | --- | --- |
| *Demographics* |  |  |  |
| Age (years) | 31.3 (13.1) | 32.4 (13.0) | 0.658 |
| Sex (% Female) | 0.58 | 0.57 | 0.909 |
| Non-binary gender (%) | 0.08 | 0.08 | 0.969 |
| *Ethnicity* |  |  |  |
| Black and Minority Ethnic (BAME) (%) | 0.45 | 0.26 | **0.043** |
| *Highest Level of Education* |  |  |  |
| High school diploma/GCSE/A-level (%) | 0.62 | 0.61 | 0.924 |
| Bachelors degree (%) | 0.32 | 0.36 | 0.731 |
| Postgraduate degree (%) | 0.05 | 0.03 | 0.543 |
| *Employment Status* |  |  |  |
| Employed (full-time or part time) or Student (%) | 0.41 | 0.88 | **<0.001** |
| *Borderline Symptoms* |  |  |  |
| Borderline Symptom List 95 | 198.1 (59.1) | 153.5 (44.2) | **<0.001** |
| Zanarini Self-Report | 18.9 (6.4) | 14.4 (4.9) | **<0.001** |

*Note.* Distribution of characteristics of BPD and Non-BPD groups. Means are displayed with standard deviations in brackets for continuous variables. *N*=38 BPD and 74 Non-BPD participants. Variables were compared with two-sample *t*-tests; P-values in bold are significant at $\alpha<0.05$.

**Table S2. Additional characteristics of the BPD sample**

| Variable | Number (%) of BPD participants (total *N*=38)  or mean score |
| --- | --- |
| *Drug and Alcohol Use* |  |
| History of harmful alcohol or substance use | 12 (31.6%) |
| *Co-morbid mental illness^*^* |  |
| Depression/anxiety | 27 (71.1%) |
| Bipolar disorder Type II | 3 (7.8%) |
| Bipolar disorder Type I | 6 (15.8%) |
| Post-traumatic stress disorder | 9 (23.7%) |
| Autistic spectrum disorder | 2 (5.3%) |
| Attention deficit hyperactivity disorder | 4 (10.5%) |
| Eating disorder | 4 (10.5%) |
| *Concomitant medications* |  |
| Any concomitant medication | 26 (68.4%) |
| Number of concomitant medications | 1.6 (1.7) |
| *Symptom Scores* |  |
| Borderline Symptom List 95 (/380) | 198.1 (59.1) |
| Zanarini Self-Report (/36) | 18.9 (6.4) |
| Clinician-rated Zanarini Scale (/36) | 15.5 (5.5) |
| Barratt Impulsivity Scale (/120) | 75.4 (16.2) |
| Hamilton Depression Rating Scale (/50) | 8.45 (5.89) |
| Young Mania Rating Scale (/60) | 4.30 (2.38) |

*Note.* Additional characteristics of BPD group. *Additional past or present diagnoses reported by participants, not assessed clinically during the study. Means are displayed with standard deviations in brackets for continuous variables.

**Table S3. Linear Regression Model of Predictors of Idiocentric Updating (**$\boldsymbol{\lambda}_{\boldsymbol{Self}}\boldsymbol{)}$

| *Independent Variable* | $\beta$ | *Standard*  *Error* | *t(82)* | *p* | $\beta$ *95% CI Lower* | $\beta$ *95% CI Upper* |
| --- | --- | --- | --- | --- | --- | --- |
| Intercept | 0.1000 | 0.2170 | 0.4609 | 0.6461 | -0.3317 | 0.5317 |
| Borderline Symptom List-95 | 0.0014 | 0.0005 | 2.7271 | **0.0078** | 0.0004 | 0.0024 |
| Depressive Symptoms | -0.0025 | 0.0013 | -1.9190 | 0.0585 | -0.0050 | 0.0001 |
| BIS-11 | -0.0021 | 0.0024 | -0.8869 | 0.3777 | -0.0068 | 0.0026 |
| Manic Symptoms | -0.0001 | 0.0019 | -0.0664 | 0.9472 | -0.0039 | 0.0036 |
| Age | 0.0029 | 0.0021 | 1.4208 | 0.1592 | -0.0012 | 0.0071 |
| BAME | 0.0311 | 0.0585 | 0.5326 | 0.5957 | -0.0852 | 0.1475 |
| Female | -0.0251 | 0.0572 | -0.4378 | 0.6627 | -0.1389 | 0.0888 |
| Non-binary gender | -0.1150 | 0.1049 | -1.0966 | 0.2760 | -0.3236 | 0.0936 |
| Education | -0.0661 | 0.0474 | -1.3934 | 0.1673 | -0.1604 | 0.0282 |
| Employed | -0.0561 | 0.0624 | -0.8991 | 0.3712 | -0.1802 | 0.0680 |

*Note.* Coefficients from linear regression model of idiocentric updating ($\lambda_{Self})$,

as a function of demographic and symptom variables, across both BPD and Non-BPD groups. A positive coefficient implies that a variable has a positive relationship with idiocentric updating. P-values in bold are significant at $\alpha<0.05$. Total *N*=112.
