# Supplementary figures and images for: A computational signature of self-other mergence in Borderline Personality Disorder
Source: Transl Psychiatry. 2024 Nov 19;14:473. doi: 10.1038/s41398-024-03170-w (PMC11576885; doi:10.1038/s41398-024-03170-w)

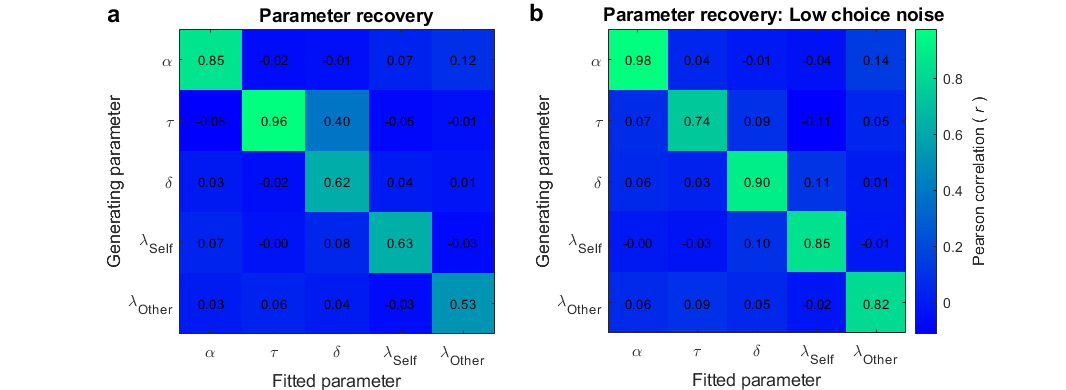

Supplement: Supplementary file 2 — Supporting Figure 1 [file 41398_2024_3170_MOESM2_ESM.tif]

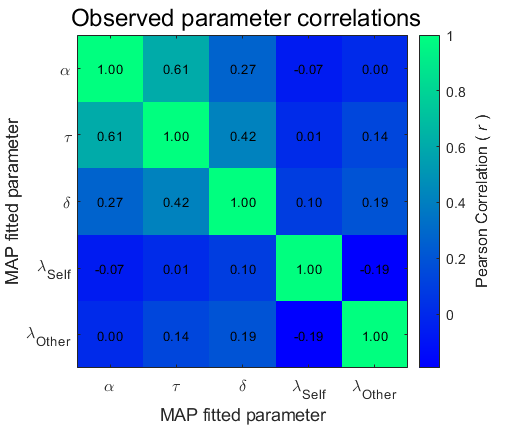

Supplement: Supplementary file 3 — Supporting Figure 2 [file 41398_2024_3170_MOESM3_ESM.tif]
